# Rapid characterization of biotherapeutic proteins by size-exclusion chromatography coupled to native mass spectrometry 

**DOI:** 10.1080/19420862.2015.1122150

**Published:** 2015-12-10

**Authors:** Markus Haberger, Michael Leiss, Anna-Katharina Heidenreich, Oxana Pester, Georg Hafenmair, Michaela Hook, Lea Bonnington, Harald Wegele, Markus Haindl, Dietmar Reusch, Patrick Bulau

**Affiliations:** Pharma Technical Development Analytics, Roche Diagnostics GmbH, Nonnenwald 2, 82377 Penzberg, Germany

**Keywords:** Aggregation, bispecific antibodies, critical quality attributes, fragmentation, native mass spectrometry, size-exclusion chromatography, ultra high performance liquid chromatography

## Abstract

High-molecular weight aggregates such as antibody dimers and other side products derived from incorrect light or heavy chain association typically represent critical product-related impurities for bispecific antibody formats.

In this study, an approach employing ultra-pressure liquid chromatography size-exclusion separation combined with native electrospray ionization mass spectrometry for the simultaneous formation, identification and quantification of size variants in recombinant antibodies was developed. Samples exposed to storage and elevated temperature(s) enabled the identification of various bispecific antibody size variants. This test system hence allowed us to study the variants formed during formulation and bio-process development, and can thus be transferred to quality control units for routine in-process control and release analytics. In addition, native SEC-UV/MS not only facilitates the detailed analysis of low-abundant and non-covalent size variants during process characterization/validation studies, but is also essential for the SEC-UV method validation prior to admission to the market.

## Introduction

Recombinantly expressed antibodies have become one of the most important therapeutic treatment options for a variety of severe diseases, and more than 40 monoclonal antibody (mAb) products have been approved by health authorities in the past 30 y^1,2^ To date, 18 bispecific antibody products are in the development pipeline of the pharmaceutical industry.^3^ Bispecific mAb production is intrinsically associated with more complex production processes compared to standard IgG molecules, and a variety of different technologies (e.g., CrossMAb design) are applied for their efficient production.^4,5^ During the production of these complex bio-molecules in living cells, numerous process- and product-related side products are generated, and these must be sufficiently removed, or at least reduced to minimal levels, to ensure maximum patient safety.

High-molecular weight aggregates such as mAb dimers and side products derived from incorrect light or heavy chain association typically represent critical product-related impurities for bispecific mAb formats. These size variants require close monitoring because they can cause immunogenic responses, or may have differences in pharmacokinetics or potency compared to the desired product.^6,7^ For the assessment of product size variants, size-exclusion-HPLC (SE-HPLC) is generally the method of choice for routine product testing in quality control laboratories. SE-HPLC is a robust method for detecting and quantifying high-molecular aggregates, and also for determining the content of low molecular forms such as product fragments.^8^ A drawback of SE-HPLC is that it does not enable accurate determination of the molecular mass of an analyte. The application of a multi-angle light scattering detector with moderate mass accuracy is commonly utilized to partially overcome this limitation.^9^

Alternatively, advances in native mass spectrometry have enabled the analysis of intact protein and protein complexes under more physiologically representative conditions.^10,11^ During recent years, several authors have successfully demonstrated the application of native MS for the qualitative and quantitative structural characterization of recombinant antibodies and new therapeutic protein formats.^12-19^ Moreover, native MS also allows the analysis of antibody oxidation, dimer formation, antibody aggregation, and antibody-antigen binding.^20-23^ In this study, we describe the development of a widely applicable, SE-UPLC-MS-based characterization method for mAbs, using an in-house bispecific antibody (CrossMAb) as model analyte.

For our study, an approach employing elevated temperature stress conditions and SE-UPLC separation combined with native MS for the simultaneous identification and quantification of size variants in recombinant antibodies was developed. This test system enabled us to study the presence and removal of critical bispecific mAb size variants at an early bioprocess development stage.

## Results

**Figure 1.**
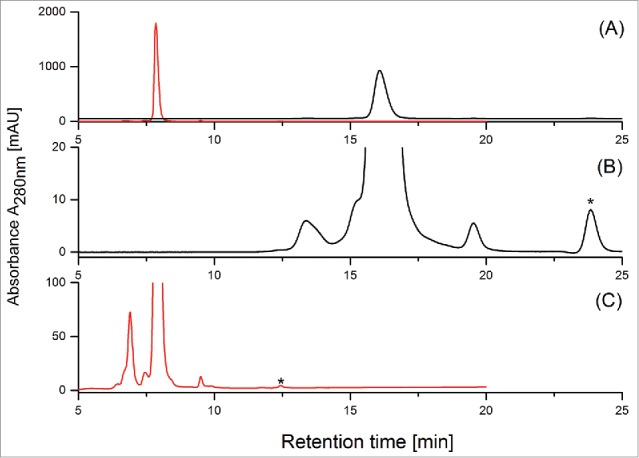
(**A**): Full scale overlay of standard HPLC-SEC (black) and FastSEC (red) chromatograms of CrossMAb reference material. Zoom-in of CrossMAb stability sample stored for 24 months at 5°C applying (**B**) standard HPLC-SEC and (**C**) FastSEC. Note: Signals marked with asterisks (*) are caused by the sample matrix.

The aim of this study was to develop a size exclusion-based ultrahigh-pressure liquid chromatography (UHPLC-SEC) method (Fast-SEC) to analyze the aggregate and fragment formation of a bi-specific CrossMAb during bio-process and formulation development. Flowrate, protein load, column temperature, ionic strength and composition of the eluents were optimized to achieve the most suitable conditions for the separation of the CrossMAb monomer from its product-related impurities (data not shown; protocol summarized in material and methods). Fig. 1A illustrates the differences in run-time and resolving power of the Fast-SEC method compared to our in-house standard HPLC-SEC release method using CrossMAb reference material. In addition to the (significantly) earlier elution time of the CrossMAb monomer (6 min versus 16 min) the Fast-SEC approach (Fig. 1C) also demonstrated superior separation power compared to the platform HPLC-SEC approach (Fig. 1B). The difference is particularly pronounced at the edges of the CrossMAb monomer peak, where improved separation of high molecular weight (HMW) and low molecular weight (LMW) species was achieved using the Fast-SEC approach.

**Figure 2.**
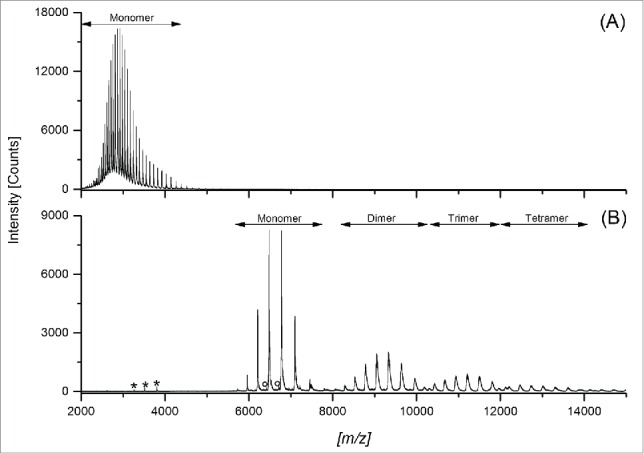
Comparison of (**A**) offline CrossMAb ESI-MS using denaturing conditions versus (**B**) offline native ESI-MS analysis. Signals for the LC_x_LC_y_ heterodimer (**Table 1**, Peak 7) are marked with an asterisk (*). Signals corresponding to the CrossMAb w/o LC_y_ + LC_x_/LC_y_ heterodimer (**Table 1**, Peak 4) are annotated with a degree sign (°). Signals corresponding to the CrossMAb w/o LC_y_, singly LC_x_, and singly LC_y_ were only detected under denaturing conditions (A; not marked due to insufficient separation from monomer signals).

**Figure 3.**
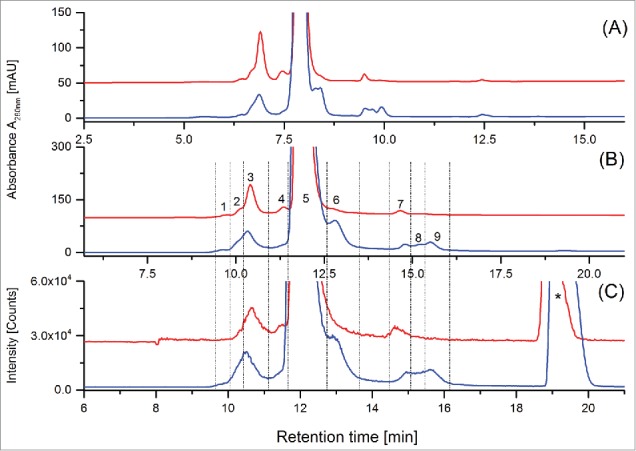
Comparison of Fast-SEC with UV detection (**A**) vs. native ESI-UV/MS (B, UV trace; C, total ion current chromatogram). Chromatograms show CrossMAb ‘stability’ (red trace, 24 months at 5°C) and temperature stressed (blue trace, 3 months at 40°C) samples. Proposed assignments of the Native ESI-MS fractions 1–9 (**B**) are summarized in Table 1. Sample matrix signals are marked with asterisk (*). The differences in retention times is due to the different flow rates applied for A (300 µl/min and B/C (200 µl/min).

In order to identify fragment and aggregate variants, the CrossMAb reference material was initially analyzed by intact electrospray ionization mass spectrometry (ESI-MS) using denaturing conditions and by native ESI-MS (Fig. 2). By the application of ESI-MS under denaturing conditions, only charge state signals corresponding to the CrossMAb monomer were detected, whereas using native (ESI-MS) conditions the CrossMAb dimer, trimer, and tetramer could also be verified, suggesting that only native ESI-MS enables detection of the full set of covalent and non-covalent CrossMAb size variants (Fig. 2; Table 1). Analysis of CrossMAb reference material by UHPLC-SEC with salt gradient and UV detection (Fig. 1A) showed minimal dimer and trimer levels (< 1%, data not shown). In contrast, analysis of the same sample by offline native ESI-MS analysis showed significant (method-induced) CrossMAb dimer, trimer and tetramer formation (Fig. 2B). In our experience, offline (direct infusion) analysis often leads to the formation of oligoform artifacts due to the high concentration in the electrospray ion-source. Furthermore, the application of native SEC-UV/MS did not suggest significant dimer and trimer formation (Fig. 3B and C). Subsequently, we aimed to characterize the LMW and HMW variants observed in the Fast-SEC UV-profile by coupling UHPLC-SEC to native ESI-MS. To perform native ESI-MS experiments, the SEC mobile phase was replaced with ammonium acetate buffer and tested in a concentration range of 25 to 100 mM. Optimal protein load was also investigated. An isocratic elution with 75 mM ammonium acetate and a protein load of 150 µg resulted in separation quality comparable to the Fast-SEC method (Fig. 3). Fig. 3 illustrates the improved resolving power of the Fast-SEC procedure (Fig. 3A) compared to the native SEC-UV/MS system (Fig. 3B) for stability samples (stored for 24 months at 5°C, red traces) and temperature stressed material (stored for 3 months at 40°C, blue traces). The recorded chromatograms demonstrate comparable separation power and detectability of non-covalent aggregates.

**Table 1.** Native ESI-UV/MS identification results of CrossMAb size variants detected in stability samples (elution profile shown in Fig. 3). HC_x_ = heavy chain x, HC_y_ = heavy chain y, LC_x_ = light chain x, LC_y_ = light chain y; *, cysteinylation (plus 119 Da) or glutathionylation (plus 305 Da) of heavy chain in position 233 (HC-Cys-233). ° indicates multiple cleavage sites of the upper hinge region, resulting in the corresponding Fc- and Fab-fragments (HC_x_/LC_x_ and HC_y_/LC_y_) (Peaks 6, 8, and 9). Identity of covalently modified (Peak 4) or truncated CrossMAb variants (Peaks 6–9) were additionally confirmed by LysC peptide mapping. n.d., not detectable.

SEC-Peak	Protein variant	Description	Charge state	Theoretical molecular mass [Da]	Reference sample [Da]	Stability sample 5°C 24 months [Da]	Stress sample 40°C 3 months [Da]
1	Trimer	Trimer of CrossMAb	35–44	447146	n.d.	447651	447573
2 + 3	Dimer	Dimer 1/2 of CrossMAb	28–36	298097	298108	298131	298108
4		CrossMAb w/o LCy + LCx/LCy Heterodimer	21–27	172595_(+Cys)_ 172782_(+GSH)_	172611 172792	172619 172800	172594 172775
5		CrossMAb	19–25	149049	149043	149052	149044
		CrossMAb w/o LC_y_	19–21	126918_(+Cys)_ 127104_(+GSH)_	126919 127107	126933 127113	126911 127100
6		CrossMAb w/o Fab HC_y_/LC_y_	16–20	100327_[HCy_240–241 H-T]_ 100693_[HCy_237–238 D-K]_ 100808_[HCy_236–237 C-D]_	100329n.d.100810	100334n.d.100810	100326100693100806
		CrossMAb w/o Fab HC_x_/LC_x_	16–20	101020_[HCx_228–229 K-T]_ 101164_[HCx_227–228 D-K]_	n.d.n.d.	n.d.n.d.	101012101165
7		Homodimer LC_x_/LC_x_	11–14	46856	46856	46858	46854
		Heterodimer LC_x_/LC_y_	11–14	45678	45677	45679	45676
8		Fab HC_y_/LC_y_	11–14	48258_[HCy_236–237 C-D]_ 48355_[HCy_237–238 D-K]_ 48739_[HCy_240–241 H-T]_	n.d.n.d.	n.d.n.d.	4826048356
					n.d	48741	48737
9		Fab HC_x_/LC_x_	12–14	47885_[HCx_228–229 D-K]_ 48029_[HCx_227–228 K-T]_	n.d.n.d.	n.d.48033	4788248028

In addition to UV detection, the native SEC-UV/MS system offers accurate online mass determination. The proposed CrossMAb aggregates (assigned Peaks 1–4) were detected over a m/z range of 8000 to 14000, the CrossMAb main monomer structure (Peak 5) between m/z 5500 to 7500 and the CrossMAb fragments (Peaks 6–10) were traceable between m/z 3000 and 6500. All selected charge states for mass determination are summarized in Table 1. Interestingly, the SEC-UV trace (Fig. 3B) and the corresponding online SEC-MS total ion current chromatogram (TIC; Fig. 3C) were similar with respect to signal pattern and relative intensities. The peak assignment is as follows: Peak 1 was identified as the CrossMAb trimer, whereas the mass determination of Peaks 2 and 3 suggest the presence of isomeric CrossMAb dimers with identical mass values in both fractions. Mass determination of Peak fraction 4 suggested a modified 3-fourths (¾, related to chain composition) CrossMAb monomer (modified by the addition of a cysteine (+119 Da) or glutathione (+305 Da) group in place of the missing LC_y_ chain) non-covalently associated with a LC heterodimer (see proposed description in Table 1). The covalent addition of cysteine or glutathione at the heavy chain y (HC_y_) cysteine in position 233 was confirmed by LysC peptide mapping using non-reductive conditions (Fig. S1 A and B). It is likely that the presence of these modifications prevented the covalent association of the expected LC_y_, thus resulting in the formation of the modified ¾ CrossMAb. The additional non-covalent association of the modified ¾ CrossMAb with a LC heterodimer (LC_x_/LC_y_) was also confirmed by LysC peptide mapping (Fig. S1 C). In the downward right flank of the CrossMAb monomer (Peak 5), the presence of traces of the modified ¾ CrossMAb with slightly shifted retention time were confirmed by native ESI-MS. Chromatographic Peak 6 could be assigned to various truncated CrossMAb variants lacking one Fab fragment as previously reported in the literature.^24^ Peak 7 masses are consistent with CrossMAb light chain homodimer (LC_x_/L_x_) and heterodimer (LC_x_/LC_y_) structures. Peaks 8 and 9 are composed of the free Fab fragments (HC_x_/LC_x _and HC_y_/LC_y_) of the corresponding truncated CrossMAb species variants detected in Peak 6. Singly modified LC_x_and LC_y_ have only been detected in trace amounts at a retention > 16 min in the extracted ion current chromatogram of the ESI-MS approach (Fig 3B). However, UV-signals of these species were not detected. The LC dimerizes preferentially resulting in the absence of singly LC. This phenomena is most likely a consequence of the applied CrossMAb technology. The CrossMAb light chain homo- and heterodimers were confirmed by LysC peptide mapping using non-reductive conditions (data not shown). Compared to the CrossMAb reference material (Fig. 1A), significant dimer formation could be detected after storage for 24 months at 5°C (Figs. 3A and B; Peak 2+3), whereas accelerated temperature (3 months at 40°C) induced predominantly truncated CrossMAb variants (Figs. 3A and B; Peaks 6, 8, and 9).

**Figure 4.**
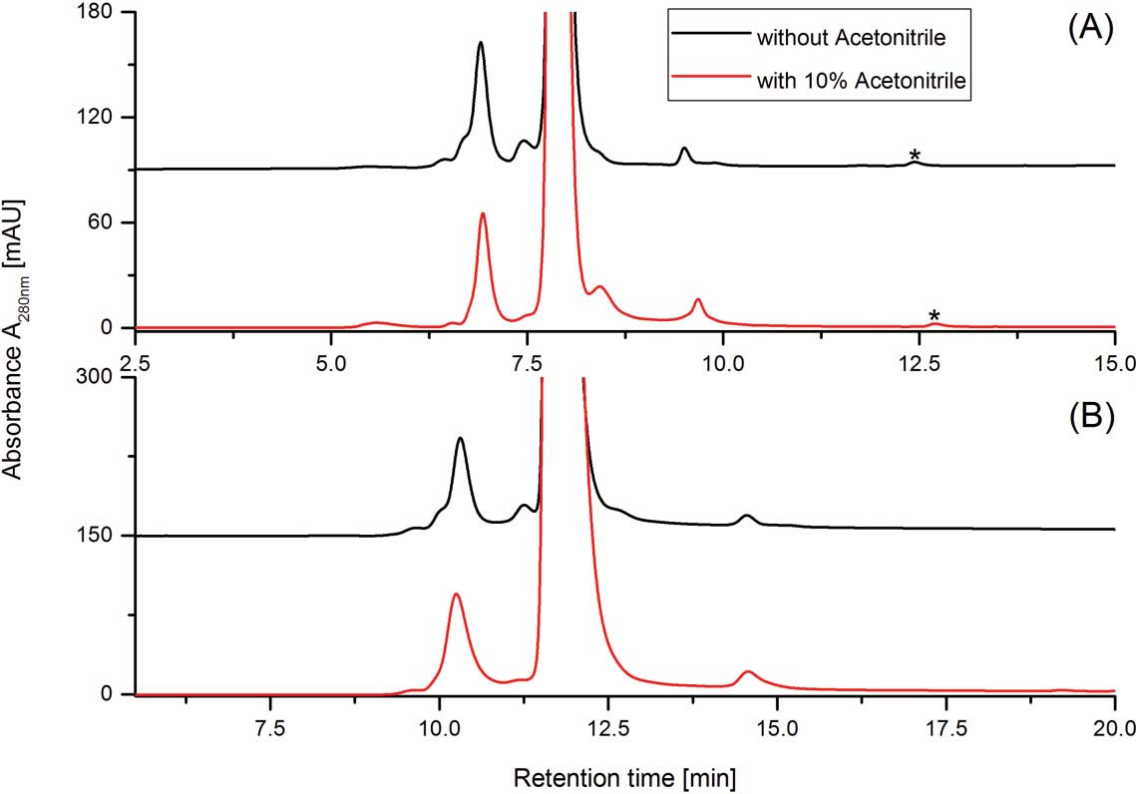
Confirmation of non-covalent CrossMAb size variants in stability samples (stored for 24 months at 5°C) by Fast-SEC with UV detection (**A**) versus native ESI-UV/MS (B, UV trace). Sample matrix signals are marked with an asterisk (*).

The mass spectrometric analysis of the CrossMAb size variants by native SEC-UV/MS suggested a high HMW species (Fig. 3B; Peak 4) consisting of a modified ¾ CrossMAb non-covalently associated with a LC-Dimer. In order to confirm this non-covalent interaction, 10% acetonitrile was added the chromatographic eluent. Fig. 4A shows the result of Fast-SEC separation with UV detection of a CrossMAb stability sample (24 month at 5°C) with and without addition of acetonitrile to the eluent. The same experiment was performed applying the SEC-UV/MS protocol, the corresponding chromatograms are given in Fig. 4B. The achieved results clearly demonstrate that addition of 10% acetonitrile induces a dissociation of the LC-Dimer from the modified ¾ CrossMAb, resulting in a decrease of the corresponding chromatographic Peak 4 and a simultaneous increase of the LC-Dimer Peak 7 (the corresponding increase in the released modified ¾ CrossMAb structure is not discernible due to coelution with the main peak. Taken together, this data demonstrates that both Fast-SEC with UV detection and native SEC-UV/MS are capable of the stabilization, separation and detection of non-covalently associated size variants derived from CrossMAb stability samples.

**Figure 5.**
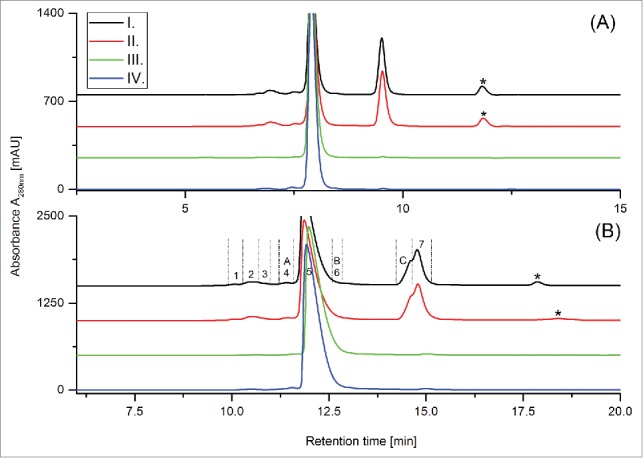
Overlay of Fast-SEC (A) and native ESI-UV/MS chromatograms (B, UV trace) of 4 in-process samples. I. Affinity purification eluate; II. Hydrophobic interaction chromatography eluate; III. Ion exchange chromatography eluate; IV. Eluate UF/DF diafiltration step. Sample matrix signals are marked with asterisk (*).

Next, we aimed to apply the Fast-SEC with UV detection and native SEC-UV/MS methods to in-process samples derived from CrossMAb bio-process development studies. Samples from the initial affinity purification step, 2 subsequent chromatographic purification steps and the final ultrafiltration/diafiltration (UF/DF) step were analyzed with both methods. The resulting native SEC-UV chromatograms are depicted in Fig. 5. Both methods demonstrated their suitability to monitor the removal of CrossMAb size variants by the developed purification process, and showed comparable chromatographic resolution and quantification results (Table S2). After the first and second purification steps, a significant amount of unbound LC and aggregates were still detectable; however, these were almost completely removed in the third purification step.

In addition, we detected new CrossMAb size variants in the bio-process samples by native SEC-UV/MS that were not traceable in the CrossMAb reference and stability material. In detail, supplementary to the already identified CrossMAb size variants (Peaks 1–9, Fig. 3B and Table 1), 3 new size variants (named A, B, and C) were identified by the native SEC-UV/MS method (Fig. 5B, Table 2). These new non-covalent CrossMAb size variants were not fully separated by the Fast-SEC with UV detection (Fig. 5A).

**Table 2.** Native ESI-UV/MS identification results of CrossMAb size variants detected in bio-process samples (elution profile shown in Fig. 5). HC_x_ = heavy chain x, HC_y_ = heavy chain y, LC_x_ = light chain x, LC_y_ = light chain y; *, cysteinylation (plus 119 Da) or glutathionylation (plus 305 Da) of light chain in position 214 (LC-Cys-214). n.d., not detectable.

SEC-Peak	Protein variant	Description	Charge state	Theoretical molecular mass [Da]	PS I [Observed mass, Da]	PS II [Observed mass, Da]	PS III [Observed mass, Da]	PS IV [Observed mass, Da]
A		CrossMAb + Heterodimer LC_x_LC_y_	23–29	194726	194725	194714	194712	194722
B		Aggregate of Heterodimer LC_x_LC_y_	17–20	91356	91356	91353	n.d.	n.d.
C		LC_x_LC_x(+2Cys)_ LC_x_LC_x(+2GSH)_ LC_x_LC_x(+Cys+GSH)_	11–14	47096 47469 47282	47096 47466 47280	47094 47464 47278	47092 n.d. 47278	47095 n.d. 47280

The size variant A corresponds to the CrossMAb monomer plus a light chain heterodimer (LC_x_/LC_y_). Size variant B was verified as the dimer of 2 light chain heterodimers (LC_x_LC_y_). Variously modified LC_x_LC_x_ homodimer variants were also detected and are summarized as size variants C. In detail, the homodimers are assigned to LC_x_LC_x_ plus either 2 disulfide bridged cysteines (2*+119 Da), 2 glutathione groups (2*+305 Da) or one cysteine and one glutathione (Table 2). The modifications are attached at the LC_x_-Cys-214 as confirmed by LysC peptide mapping (data not shown). In summary, Fast-SEC with UV detection and native SEC-UV/MS both represent suitable methods to study the formation and removal of critical CrossMAb size variants in bioprocess development.

## Discussion

For characterization of CrossMAb bio-therapeutics, a new approach employing fast size-exclusion chromatography (SEC) with UV detection coupled online to native electrospray ionization mass spectrometry was developed (native SEC-UV/MS) because, using our in-house standard HPLC-SEC method (developed for the release and stability testing of therapeutic antibodies), resolving CrossMAb size variants sufficiently was not adequate. In a recently published study, the successful application of UHPLC-SEC with 2 µm particle size for testing of IgG1 product-related impurities was reported.^25^ In our study, 2 complementary UHPLC-SEC test procedures for the detailed characterization of CrossMAb aggregates and fragments were developed. For the fast characterization of CrossMAb samples derived from formulation and bio-process development, an UHPLC-SEC test system with salt gradient and UV detection (Fast-SEC) was optimized. The Fast-SEC approach demonstrated improvements to run time and chromatographic separation resolving power compared to our in-house standard HPLC-SEC approach and was suitably robust for the analysis of CrossMAb size variants derived from stress stability and bio-process development studies. Multi-angle light scattering is commonly used to analyze the molecular mass composition of SEC peak fractions during biopharmaceutical method development and validation. The limitation of this approach, however, is the low resolution and moderate mass accuracy, particularly when low abundant aggregate species are the subject of investigation.^9,26^

Thus, we aimed to establish an online SEC-UV/MS system for the direct mass spectrometric characterization of CrossMAb size variants without prior preparative SEC fractionation. We previously described the optimization of mass spectrometric instrument parameters for functional assessment of non-covalent antibody/receptor complexes by native mass spectrometry.^21^ Initial characterization of CrossMAb reference material by offline intact (using organic solvent and formic acid as the eluant) and native ESI-MS demonstrated that only under native ESI-MS buffer conditions are non-covalent CrossMAb size variants such as dimers, trimers and tetramers adequately stabilized, but also artificially induced, in subsequent mass spectrometric detection. It is important to note that care must be taken to ensure the species observed are real and not artificially induced in the electrospray process. These results are in agreement with previous studies on the stabilization/characterization of antibody complexes by native ESI-MS.^20-23^

An optimized native SEC-UV/MS protocol using 75 mM ammonium acetate as elution solvent yielded separation quality comparable to the Fast-SEC test system and adequate signal intensity for mass detection by ESI-MS. The proposed CrossMAb trimer and dimer aggregates were chromatographically separated from the abundant CrossMAb monomer signal. Moreover, a modified ¾ CrossMAb monomer (lacking LC_y_; addition of cysteine or glutathione) non-covalently associated with a LC heterodimer (LC_x_/LC_y_) aggregate variant was found to elute closely to the main monomer peak. This so far undescribed CrossMAb aggregate variant was supplementary confirmed by the addition of organic solvent to the eluent (to confirm the non-covalent nature of interaction) and by LysC peptide mapping of collected SEC fractions using non-reductive conditions (to locate the covalent cysteine or glutathione modification). We identified that both modifications occur at heavy chain Cys-233, indicating that this variant is not able to covalently bind their respective LC_y_. Cysteinylation or glutathionylation of free light chain cysteines has been reported in a previous study;^27^ however, modification of heavy chain residues has, to our best knowledge, not been described so far.

Trace amounts of modified ¾ CrossMAb monomer alone were verified to co-elute with the abundant CrossMAb monomer signal, and thus the species was not quantifiable by Fast-SEC with UV detection. Various truncated CrossMAb species, LC homo-/heterodimers, and modified free LC variants were detected to partially co-elute in the lower molecular weight region of SEC-UV/MS separation. It should be noted that mass accuracy for the online SEC-MS peak identification with the utilized Q-TOF Ultima mass spectrometer ranged from ∼5 ppm to ∼1000 ppm. As expected, mass accuracy for the high-molecular aggregate variants was intrinsically lower compared to the abundant CrossMAb monomer and the low-molecular fragment variants (due to lower signal intensity and resolution at higher *m/z* values). Thus, where possible, the identity of covalently modified or truncated CrossMAb variants was additionally confirmed by LysC peptide mapping.

Moreover, we detected new CrossMAb size variants in bio-process derived samples with native SEC-UV/MS, which were not traceable in the CrossMAb reference and (stressed) stability material. Due to the relative similar mass of the CrossMAb size variants formed under temperature stress conditions (Table 1; Peaks 4, 6, and 7) vs. the new size variants discovered in the bio-process intermediate stage samples (Table 2; Peaks A, B, and C), the new variants were only verifiable by native SEC-UV/MS, with the Fast-SEC with UV detection method lacking sufficient resolution (Fig. 5). In conclusion, peak assignment during SEC method development for in-process control analysis should not only rely on qualitative comparison of SEC-UV chromatograms, but should also be verified by native online ESI-MS experiments.

To summarize, our results demonstrate that SEC with UV detection and native ESI-MS represent complementary test systems for the analysis of various CrossMAb aggregate and fragment variants. SEC with UV detection facilitates fast and robust analysis, especially of non-covalent CrossMAb interactions. The coupling of SEC-UV to native ESI-MS not only allows the stepwise identification of abundant CrossMAb size variants (like dimers or free LC) by accurate mass determination, but also enables the enrichment and characterization of various low-abundant and non-covalent aggregate and fragment variants. Optimized native ESI-MS spray conditions and instrument settings represent a compromise between stabilization and artificial formation of protein complexes.^21^ Thus, the comparison of SEC-UV and SEC-MS data is needed to identify experimental artifact aggregate or fragment formation in the ion source of the applied MS system. Taken together, the developed Fast-SEC system is suitable to monitor various CrossMAb size variants during formulation and bio-process development, and can thus be transferred to quality control units for routine in-process control and release analytics. In addition, native SEC-UV/MS not only facilitates the detailed analysis of low-abundant and non-covalent size variants during process characterization/validation studies, but is also essential for the SEC-UV method validation prior to admission to the market. The reported native SEC-UV/MS methodology and results might also be of importance for studying antibody-antigen interactions and for other major classes of biopharmaceuticals such as Fc-fusion proteins and protein scaffolds.^12,28^

## Materials and methods

### Offline ESI-MS analysis

Offline ESI-MS analysis of CrossMAb samples was performed on a modified Q-TOF Ultima mass spectrometer system (Waters Corp., Manchester, UK) upgraded by MS Vision (Almere, The Netherlands) as a High Mass QTOF enabling measurement of protein/protein complexes at higher *m/z* ranges. Samples were either buffer exchanged into denaturing electrospray medium (1% formic acid in 40% acetonitrile/water; v/v) or analyzed under native MS conditions using 75 mM ammonium acetate buffer at pH 6.0 using NAP™-5 gel filtration columns. Prepared samples were introduced into the MS system using the NanoMate® direct infusion system TriVersa (Advion, Ithaca, NY, USA). As previously described, optimized MS parameters were used, which allowed adequate detection of non-covalent protein/protein complexes.^21^ Briefly, cone voltage was set at 45 V, RF Lens1 at 150 V and collision energy to 20 V. The vacuum in the collision cell was adjusted to 1.10 e^−2^ mbar. Additionally, the source vacuum was set to 2.5–2.7 bar resulting in vacuum values for the mass analyzer of around 1.42 e^−4^ and 7.42 e^−7^ for the TOF Penning.

### LysC peptide mapping using non-reductive conditions

For the detection and quantification of modifications like cysteinylation or glutathionylation at peptide level, 250 µg of CrossMAb was made up to 300 µL with 0.1 M sodium acetate, 8 M guanidine-HCl, 50 mM *N*-ethylmaleimide pH 5.0 solution and incubated for 1h at 55°C for denaturation and capping of potential free thiols. Next, the buffer solution was exchanged to a digestion buffer (0. 1 M Tris-HCl, pH 7.0) using NAP™-5 gel filtration columns. The NAP™-5 eluate (500 µL) was mixed with 70 µL of a 0.3 mg/mL Lys-C solution (Roche Diagnostics GmbH, Penzberg, Germany) and incubated at 37°C for 18 hours.

### Analysis of proteolytic LysC peptides by liquid chromatography mass spectrometry

The LysC peptide mixture was separated by RP-UPLC® (ACQUITY, Waters, Manchester, UK) on a C18 column (BEH C18 1.7 µm, 2.1×150 mm; Waters) and analyzed online with a Synapt G2 QTOF electrospray mass spectrometer (Waters). The mobile phases consisted of 0.1% formic acid in water (solvent A) and 0.1% formic acid in acetonitrile (solvent B). The chromatography was carried out using a gradient from 1 to 35% solvent B in 45 min and finally from 35 to 80% solvent B in 3 min using a flow rate of 300 µL/min. UV absorption was measured at a wavelength of 220 nm. A sample amount of 3.5 µg digested protein was applied. The UPLC system and mass spectrometer were connected by PEEK capillary tubing. Data acquisition was controlled by the MassLynx™ software (Waters). Parameters for MS detection were adjusted according to existing knowledge gained from experience with peptide analysis of recombinant antibodies.

### Data analysis for the quantification of chemical modification levels

Peptides of interest were identified by searching manually for their *m/z* values within the mass spectrum. For the quantification, specific ion current (SIC) chromatograms of peptides of interest were generated on the basis of their monoisotopic masses and detected charge states using the in-house written MassMap® software module, created within the GRAMS AI software (Version 8.0, Thermo Scientific, Dionex Softron GmbH, Germering, Germany).^29^ The relative amounts of CrossMAb modifications were calculated from the manual integration results of the modified and unmodified peptide peaks.

### Size-exclusion chromatography directly coupled to native ESI-MS (native SEC-UV/MS)

The native SEC-UV/MS was carried out using an ACQUITY UPLC® Protein BEH SEC column (4.6 × 300 mm, 1.7 µm particle size; Waters, Milford, MA, USA). An isocratic elution using 75 mM CH_3_COONH_4_, pH 6.0 at 0.2 mL/min was used for chromatographic separation on a Dionex UltiMate® 3000 RSLC-system (Thermo Scientific, Dionex Softron GmbH, Germering, Germany) equipped with UV detection at 280 nm. Sample injection amounts of 150 µg mAb were used and data acquisition was controlled by Chromeleon software (Thermo Scientific, Dionex Softron GmbH, Germering, Germany). Relative quantification was achieved by manual integration of the chromatographic peaks and calculation of the ratio of the relevant peak areas. The outlet of the RSLC system was directly coupled to the NanoMate® direct infusion system (Advion, Ithaca, NY, USA). The RSLC flow was split into 2 parts, 4 µl/min were online infused to the MS system and 194 µl either automatically collected into 384-well plates or directly disposed to the waste. The NanoMate® system was installed on the afore-described modified ‘High Mass QTOF’ (see ESI-MS analysis section).

### Size-exclusion chromatography by HPLC-SEC

The standard SEC protocol was carried out using a Thermo Scientific (Dionex Softron GmbH, Germering, Germany) UltiMate® 3000 UHPLC system including autosampler, high pressure gradient solvent delivery pumps, column oven, and diode array detector (detection at 280 nm wavelength), using a TSKgel G3000SWxl SEC column (300 × 7.8 mm, 250˚A, 5µm particle size) from Tosoh Bioscience (Griesheim, Germany). Running conditions were oven temperature 25°C, flow rate 0.5 ml/min, run time 30 min and mobile phase 200 mM KH_2_PO_4_, 250 mM KCl, pH 7.0. Sample amounts of 5 µl of a 30 mg/ml CrossMAb protein solution were injected, corresponding to 150 µg on column.^30^

### Size-exclusion chromatography by UHPLC (Fast-SEC method)

The Fast-SEC protocol was carried out using a Thermo Scientific (Dionex Softron GmbH, Germering, Germany) UltiMate® 3000 Rapid Separation UHPLC system including autosampler, high pressure gradient solvent delivery pumps, column oven, and diode array detector (280 nm detection wavelength) and an ACQUITY UPLC BEH200 SEC column (300×4.6 mm, 200 Å, 1.7 µm particle size) from Waters (Milford, MA, USA). Running conditions were as follows: oven temperature 40°C, eluent flow rate 0.3 ml/min, overall run time 20 min. The mobile phase composed of 200 mM KH_2_PO_4_ and 250 mM KCl at pH 7.0. Sample amounts of 5 µl from a 12 mg/ml protein solution of the CrossMAb were injected, corresponding to 60 µg total protein amount on column. For measurements with organic solvent, 10% (v/v) of acetonitrile was added to the mobile phase.

## Supplementary Material

KMAB_A_1122150_supplemental_material.docx
