# Supplementary material for: Rapid characterization of biotherapeutic proteins by size-exclusion chromatography coupled to native mass spectrometry
Source: MAbs. 2015 Dec 10;8(2):331–9. doi: 10.1080/19420862.2015.1122150 (PMC4966600; doi:10.1080/19420862.2015.1122150)

Supplemental Table 1:

SEC-UV quantification of CrossMAb size variants in stability samples by native ESI-UV/MS (see Fig. 3)

| **SEC-Peak** | **Protein variant** | **Description** | **Reference**  **sample  [area %]** | **Stability sample 5°C 24 mths  [area %]** | **Stress sample 40 °C 3 mths  [area %]** |
| --- | --- | --- | --- | --- | --- |
| 1 | Trimer | Trimer of CrossMAb | n.d. | 0.2 | 0.2 |
| 2 + 3 | Dimer | Dimer 1/2 of CrossMAb | 0.9 | 5.5 | 4.0 |
| 4 | 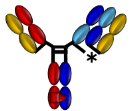 | CrossMAb w/o LC_y_, + LC_x_/LC_y_ Heterodimer | 1.1 | 1.1 | 0.7 |
| 5 | **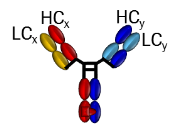** | CrossMAb | 96.1 | 91.2 | 86.2 |
|  | 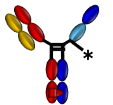 | CrossMAb w/o LC_y_ |  |  |  |
| 6 | **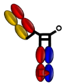** | CrossMAb w/o Fab HC_y_/LC_y_ | n.d. | n.d. | 5.1 |
|  | **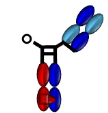** | CrossMAb w/o Fab HC_x_/LC_x_ |  |  |  |
| 7 | **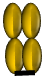** | Homodimer LC_x_/LC_x_ | 0.9 | 0.8 | 0.8 |
|  | **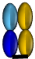** | Heterodimer LC_x_/LC_y_ |  |  |  |
| 8 | **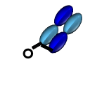** | Fab HC_y_/LC_y_ | n.d. | n.d. | 0.7 |
| 9 | **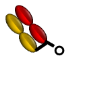** | Fab HC_x_/LC_x_ | n.d. | n.d. | 1.2 |

Supplemental Table 2:

SEC-UV quantification of aggregates and fragments in bio-process samples by Fast-SEC or native ESI-UV/MS conditions (see Fig. 5)

| Size variant | Method | Purification step (PS) | | | |
| --- | --- | --- | --- | --- | --- |
|  |  | PS I  [area %] | PS II  [area %] | PS III  [area %] | PS IV  [area %] |
| Aggregates | Fast-SEC | 4.5 | 4.1 | 0.6 | 0.7 |
|  | native ESI-UV/MS | 4.7 | 4.2 | 0.8 | 0.7 |
| Monomer | Fast-SEC | 70.6 | 70.8 | 98.8 | 98.8 |
|  | native ESI-UV/MS | 70.7 | 71.0 | 98.5 | 98.6 |
| Fragments | Fast-SEC | 24.8 | 25.1 | 0.6 | 0.5 |
|  | native ESI-UV/MS | 24.6 | 24.8 | 0.8 | 0.7 |

Supplemental Fig. 1:

LysC peptide mapping analysis of isolated SEC fractions 4 and 5 (see Table 1) using non-reductive conditions. Specific ion current chromatograms of heavy chain peptides carrying an additional cysteine (A, framed) or glutathione (B, framed) at position HCx-Cys-233 are displayed. Additionally, specific disulfide-linked peptide signals confirming the presence of LC_x_/LC_y_ heterodimer in SEC fraction 4 is shown (C, framed).


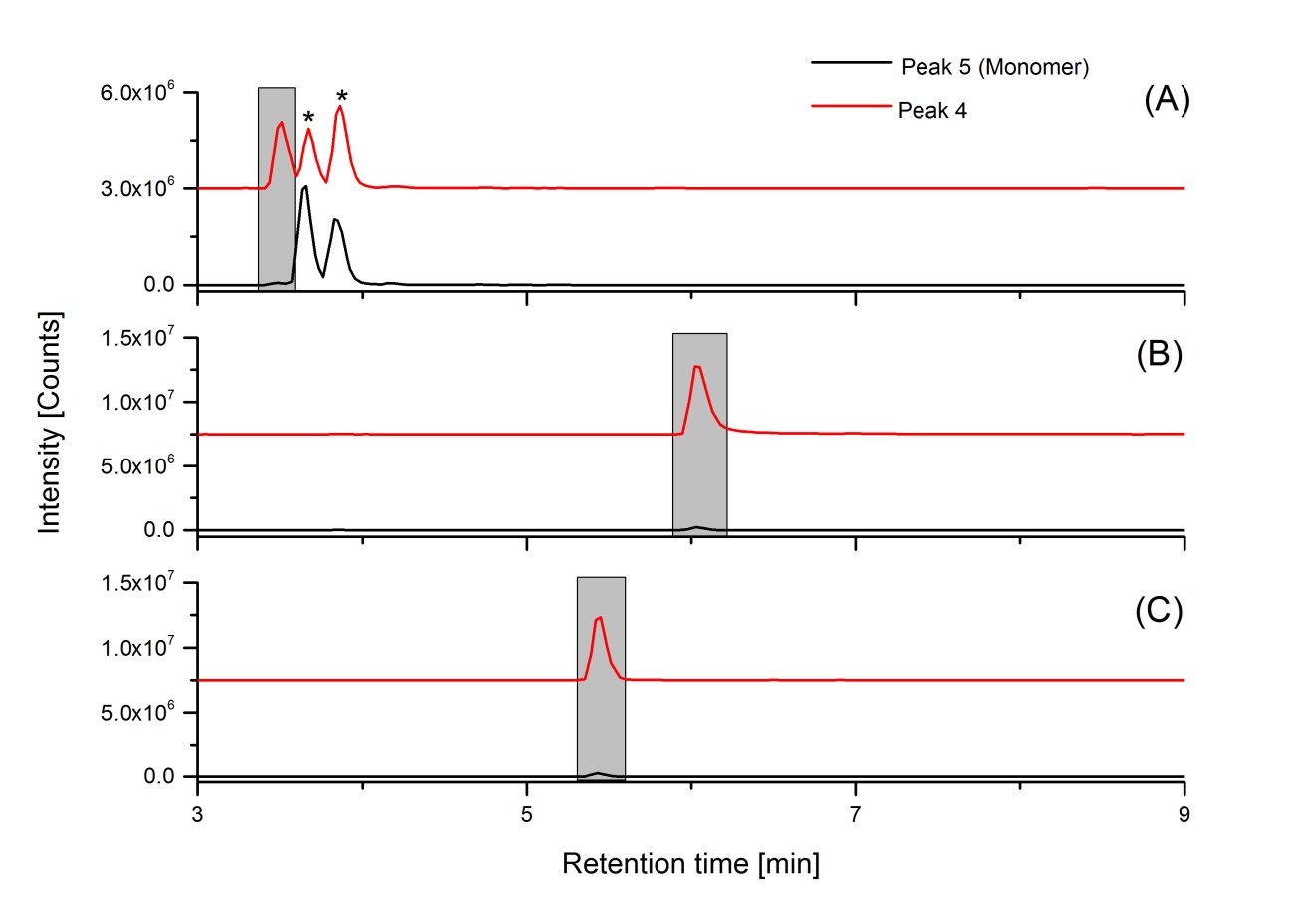

Supplement: KMAB_A_1122150_supplemental_material.docx [file kmab-08-02-1122150-s001.docx]
